# Supplementary material for: Genetic Study of SARS-CoV-2 Non Structural Protein 12 in COVID-19 Patients Non Responders to Remdesivir
Source: Microbiol Spectr. 2022 Nov 10;10(6):e02448-22. doi: 10.1128/spectrum.02448-22 (PMC9769853; doi:10.1128/spectrum.02448-22)
Supplement: Supplemental file 1 — Supplemental material. Download spectrum.02448-22-s0001.pdf, PDF file, 0.4 MB [file spectrum.02448-22-s0001.pdf]

SUPPLEMENTARY DATA

Table S1. Clinical characteristics of the patients with SARS-CoV-2 non-structural protein 12 and/or Spike mutations of interest.

ID	Age	Comorbidity	ICU admission	Mortality	Immunossuppressive treatment	Antiviral treatment	Date of infection	Linage	NSP12	
									Before RDV	After RDV
1	60	Hypertension, enolic dilated myocardiopathy	no	no	BNB	RDV 5d	March 2021	Alpha (B.1.1.7)	-	A13535G (Y32C), C14120T (P227L), C15324T
2	68	Hypertension	yes	no	TCZ, CORT	RDV 5d	April 2021	Alpha (B.1.1.7)	C14120T (P227L)	C14120T (P227L), C15324T
3	40	No	no	no	BNB	RDV 5d	March 2021	Alpha (B.1.1.7)	-	G14547A
4	47	No	no	no	TCZ, BNB, CORT	RDV 5d	April 2021	Alpha (B.1.1.7)	A13535G (Y32C), C14120T (P227L), C15324T	G14547A
5	40	No	yes	no	TCZ, BNB, CORT	RDV 5d	April 2021	Delta (B.1.167)	A13535G (Y32C), C14120T (P227L), C15324T	G14547A
6	49	Thalassemia minor	no	no	BNB	RDV 5d	June 2021	Alpha (B.1.1.7)	-	G15652T (D738Y)
7	83	Diffuse large B-cell lymphoma	yes	yes	R-CHOP[a]	LPV + RTV + HCQ 7d, AZM 5d, RDV 10d + 8d, plasma	March – December 2021	Delta (B.1.167)	-	C13551T, A13689T (E83D)
8	67	Kidney transplant, arterial hypertension, hypercholesterolemia	no	no	BNB 10d	RDV 10d	July 2021	Delta (B.1.167)	G15910T	C15237T, G15910T

9	64	Mantle lymphoma in complete remission	yes	No	DEX 10d, TCZ, BNB, anakinra	RDV 20d, TEC, IVM + plasma	August 2021	Delta (B.1.167)	-	C15952A (L838I)

[a] R-CHOP is a chemotherapy composed by the combination of rituximab, cyclophosphamide, hydroxidaunorubicine, oncovin and prednisone

Abbreviations: ICU intensive care unit, LPV lopinavir, RTV ritonavir, RDV remdesivir, HCQ hydroxichloroquine, AZM Azithromycin, TEC teicoplanin, IVM ivermectin, TCZ tocilizumab, BNB baricinitinib, DEX dexamethasone, CTX cyclophosphamide, PDN prednisone, CORT other corticoids.
